# Vision-Related Quality of Life and Work Productivity in Patients With Keratoconus and Glaucoma: A Comparative Cross-Sectional Study

**DOI:** 10.7759/cureus.114405

**Published:** 2026-08-12

**Authors:** Maria Dionysopoulou, Nikos Kotsopoulos, Eleftheria Karampli, Dimitrios S Papaconstantinou, Marilita M Moschos, Vasiliki Efthymiou, Maria Liapi, Konstantinos Droutsas

**Affiliations:** 1 First Department of Ophthalmology, School of Medicine, National and Kapodistrian University of Athens, Athens, GRC; 2 Department of Health Economics, School of Economics and Political Science, National and Kapodistrian University of Athens, Athens, GRC; 3 Laboratory for Health Technology Assessment, Department of Public Health Policy, School of Public Health, University of West Attica, Athens, GRC; 4 First Department of Ophthalmology, School of Medicine, Gennimatas Hospital, National and Kapodistrian University of Athens, Athens, GRC; 5 Center for Adolescent Medicine and Adolescent Health Care, First Department of Pediatrics, School of Medicine, National and Kapodistrian University of Athens, Athens, GRC; 6 Health Policy, Independent Authority for Public Revenue (IAPR), Athens, GRC

**Keywords:** chronic ophthalmic diseases, keratoconus, nei vfq-25, patient-reported outcomes, primary open-angle glaucoma, vision-related quality of life, work productivity, wpai

## Abstract

Background: Keratoconus and primary open-angle glaucoma (POAG) are chronic ocular diseases causing visual impairment and affecting quality of life, daily functioning, and work productivity beyond visual acuity loss. Although both cause chronic visual disability, they differ in pathophysiology, with keratoconus affecting corneal optics and POAG causing progressive optic nerve damage. Comparative evidence regarding their impact on vision-related quality of life (VRQoL) and work productivity remains limited. This study compared patient-reported outcomes between individuals with keratoconus and POAG attending a tertiary ophthalmology center in Greece.

Methods: In this comparative cross-sectional study, 153 adults (102 with keratoconus and 51 with POAG) completed the National Eye Institute Visual Function Questionnaire-25 (NEI VFQ-25) and the Work Productivity and Activity Impairment Questionnaire (WPAI). Group differences were assessed using independent-samples t-tests or Mann-Whitney U tests, as appropriate. Two-way analysis of variance (ANOVA) examined the effects of demographic and clinical factors on questionnaire outcomes. Statistical significance was defined as p < 0.05.

Results: Patients with keratoconus reported significantly better VRQoL than those with POAG, with higher scores in near and distance activities, peripheral vision, social functioning, driving, color vision, and overall VFQ-25 composite score (all p < 0.05). No significant differences were observed in general vision, ocular pain, or mental health. WPAI scores did not differ significantly between groups, indicating a comparable impact on work productivity. Two-way ANOVA showed that employment status, sex, treatment modality, and comorbidities significantly influenced selected VFQ-25 domains.

Conclusions: Keratoconus was associated with better VRQoL than POAG, potentially reflecting differences in disease mechanisms and the greater potential for visual rehabilitation through optical correction. Despite these differences, both conditions had a similar impact on work productivity, emphasizing the need to assess functional and socioeconomic outcomes in chronic ophthalmic diseases.

## Introduction

Keratoconus and primary open-angle glaucoma (POAG) are among the most clinically significant chronic ophthalmic diseases, both capable of causing substantial visual impairment and exerting profound effects on patients' daily functioning, psychosocial well-being, and health-related quality of life [1,2]. Although visual disability is a common outcome of both conditions, they differ fundamentally in terms of pathophysiology, age at onset, disease progression, therapeutic approaches, and patterns of visual dysfunction. Consequently, the nature and magnitude of the burden experienced by affected individuals may vary considerably between these conditions, highlighting the importance of evaluating patient-reported outcomes in addition to conventional clinical indicators.

Keratoconus is a progressive corneal ectatic disorder characterized by localized stromal thinning and anterior protrusion of the cornea, resulting in irregular astigmatism and progressive myopia. The disease typically manifests during adolescence or early adulthood and may continue to progress for many years before stabilizing [3,4]. Recent epidemiological evidence estimates a prevalence of approximately 289.1 cases per 100,000 individuals (0.24% of the population), with the highest prevalence observed among individuals aged 20-29 years [5,6]. Because keratoconus primarily affects individuals during their most productive educational and working years, its consequences frequently extend beyond visual dysfunction to include psychological distress, social limitations, reduced work performance, and a substantial economic burden associated with disease monitoring and treatment [7,8]. Recent qualitative studies have further highlighted the multidimensional nature of this burden, with patients reporting limitations in daily activities and social participation, emphasizing the need for individualized supportive strategies [9].

The therapeutic armamentarium for keratoconus has expanded considerably over the past decades. Corneal collagen cross-linking, intrastromal corneal ring segment implantation, rigid contact lens fitting, and penetrating or lamellar keratoplasty now provide effective options for disease stabilization and visual rehabilitation [10]. Studies employing validated vision-related quality-of-life instruments have consistently demonstrated significant impairments in visual function and patient-reported outcomes among affected individuals [11-13]. Furthermore, a recent systematic review and meta-analysis confirmed a significantly increased risk of depression among patients with keratoconus compared with individuals without ocular disease, underscoring the psychological dimension of the condition [14].

In contrast, POAG represents a heterogeneous group of progressive optic neuropathies characterized by retinal ganglion cell loss, structural damage to the optic nerve, and irreversible visual field defects. It is currently one of the leading causes of permanent blindness worldwide, affecting more than 76 million individuals globally, with prevalence projections exceeding 110 million cases by 2040 [10,13]. More recent estimates suggest that the number of individuals affected by POAG may surpass 186 million by 2060, largely owing to population aging [15]. Unlike keratoconus, glaucoma predominantly affects middle-aged and older adults and often progresses insidiously, with substantial visual field loss occurring before patients become aware of functional impairment. This asymptomatic early course frequently results in delayed diagnosis, contributing to increased disease burden and irreversible visual disability [16].

The impact of POAG extends beyond visual function alone, with glaucomatous damage being associated with reduced quality of life, functional limitations, and psychosocial burden [17-20]. For example, a study involving 201 patients with glaucoma identified strong associations between comorbidities, reduced visual acuity, and poorer quality of life, whereas longer disease duration was associated with greater psychological burden [17]. Furthermore, a systematic review and meta-analysis confirmed an increased prevalence of depressive and anxiety disorders among individuals with POAG [18]. Peripheral visual field loss has been recognized as one of the strongest predictors of reduced vision-related quality of life (VRQoL) because of its disproportionate impact on mobility, spatial orientation, navigation in unfamiliar environments, and the performance of everyday activities [21].

Alongside the growing recognition of the limitations of traditional ophthalmic outcome measures, increasing emphasis has been placed on patient-reported experiences as essential complements to clinical assessment. Structural and functional indicators, including visual acuity, intraocular pressure, corneal topographic parameters, and visual field sensitivity, provide valuable information regarding disease status. However, they do not fully capture patients' subjective functional experiences or the real-world consequences of visual impairment. The National Eye Institute Visual Function Questionnaire-25 (NEI VFQ-25) has become one of the most widely used instruments for assessing VRQoL across multiple domains, including general vision, near and distance activities, driving, color vision, peripheral vision, social functioning, mental health, role difficulties, dependency, and ocular pain [22-24].

The socioeconomic consequences of chronic ophthalmic diseases are increasingly recognized as critical outcome domains. The Work Productivity and Activity Impairment Questionnaire (WPAI) is a well-established instrument for quantifying the occupational and functional consequences of chronic disease, assessing absenteeism, reduced productivity while working (presenteeism), overall work productivity loss, and impairment in regular activities outside the workplace [25]. A recent systematic review documented the substantial impact of glaucoma on work productivity and highlighted the indirect economic burden imposed by the disease on both patients and society [26]. Incorporating work productivity measures into ophthalmic research enables a more comprehensive assessment of disease burden from both societal and patient-centered perspectives, thereby informing healthcare resource allocation and the development of targeted health policies.

Although numerous studies have evaluated quality of life in patients with either keratoconus or glaucoma, direct comparative investigations between these two distinct ophthalmic disorders remain remarkably scarce. Existing evidence is derived primarily from single-disease studies or comparisons with healthy control populations, leaving the relative burden associated with these conditions insufficiently characterized. This knowledge gap is particularly important because keratoconus primarily compromises central visual quality through refractive distortion, whereas glaucoma predominantly affects peripheral visual function through progressive optic nerve damage, potentially resulting in divergent effects on daily functioning and occupational performance [27,28].

Comparative studies of this nature are of particular clinical and epidemiological relevance because they facilitate a deeper understanding of how demographic and clinical characteristics influence patient-reported outcomes. Such evidence may contribute to the identification of high-risk subgroups vulnerable to reduced quality of life and support the development of individualized supportive interventions [29].

Therefore, the primary objective of the present study was to compare VRQoL and work productivity between patients with keratoconus and patients with primary open-angle glaucoma receiving care at a tertiary ophthalmology center in Greece, using the validated NEI VFQ-25 and WPAI.

The secondary objectives were to investigate whether patient-reported outcomes differed according to predefined demographic and clinical characteristics, including sex, employment status, treatment modality, and comorbidity status. In addition, the study aimed to examine potential interactions between disease type and these factors through subgroup analyses and two-way analysis of variance (ANOVA), in order to identify characteristics associated with greater impairment in vision-related quality of life and work productivity.

## Materials and methods

Procedure

This comparative cross-sectional study included patients diagnosed with keratoconus or POAG who attended the outpatient clinics of the First Department of Ophthalmology at the General Hospital of Athens following scheduled medical appointments between February 2023 and February 2025.

The study was conducted within the Glaucoma Unit, the Electrophysiology Unit, the Ophthalmic Emergency Department, and the specialized Cornea Service of this university-affiliated department, which serves as a tertiary ophthalmology referral center for patients primarily originating from Central and Southern Greece. Data collection was performed using self-administered questionnaires completed by participants following their routine clinical examination.

A total of 160 eligible patients were approached during the study period, of whom 153 agreed to participate and provided written informed consent (response rate: 95.6%; 102 with keratoconus and 51 with POAG), including 102 individuals with keratoconus and 51 individuals with primary open-angle glaucoma. All diagnoses had been established prior to study enrollment by experienced ophthalmologists according to standard clinical criteria.

Eligibility criteria and sampling

Eligible participants were adults aged 18 years or older with a confirmed diagnosis of either keratoconus or POAG and sufficient proficiency in the Greek language to understand and complete the study questionnaires. A small number of patients with previous glaucoma surgery were included in the study, provided they fulfilled the eligibility criteria.

Patients were excluded if they had an acute ophthalmic condition requiring urgent treatment, severe cognitive impairment, or additional ocular disorders that could substantially affect visual function and potentially confound the assessment of vision-related quality of life or work productivity. For participants with keratoconus, disease severity was classified by a cornea specialist according to the Amsler-Krumeich classification system, which categorizes keratoconus into four stages (Stages I-IV), corresponding to mild, moderate, severe, and advanced disease, respectively [30]. All glaucoma participants had a confirmed diagnosis of POAG established by experienced ophthalmologists based on routine clinical evaluation. However, glaucoma severity was not formally classified using a validated staging system, as disease staging was not part of the study design.

A convenience sampling approach was employed. All eligible patients attending the participating clinics during the study period were invited to participate. Prior to enrollment, all participants received detailed information regarding the study objectives and procedures and provided written informed consent.

Ethics approval

The study was conducted in accordance with the ethical principles outlined in the Declaration of Helsinki. Ethical approval was obtained from the Scientific Board of the General Hospital of Athens "G.Gennimatas" and from the Bioethics Committee of the Medical School, National and Kapodistrian University of Athens (ref. number 568/19-11-2021). Participant confidentiality and anonymity were strictly maintained throughout all stages of the study.

VRQoL assessment

VRQoL was assessed using the NEI VFQ-25, a widely utilized instrument in both clinical and epidemiological ophthalmic research [29]. The NEI VFQ-25 evaluates multiple domains of visual functioning and quality of life, including general vision, ocular pain, near activities, distance activities, social functioning, mental health, role difficulties, dependency, and driving.

The questionnaire has demonstrated excellent reliability and validity across diverse ophthalmic populations. Each subscale is scored on a scale ranging from 0 to 100, with higher scores indicating better visual functioning and quality of life. A score of 100 reflects the best possible status, whereas a score of 0 represents the poorest possible outcome. The instrument has been culturally adapted and validated for use in the Greek population and is freely available for research purposes [31].

Work productivity assessment

The impact of keratoconus and POAG on occupational performance and daily functioning was assessed using the WPAI. The WPAI measures four domains: absenteeism (work time missed due to health problems), presenteeism (reduced productivity while working), overall work productivity loss (a composite measure of absenteeism and presenteeism), and impairment in regular non-work-related activities [32].

Higher WPAI scores indicate greater impairment and poorer outcomes across all domains, reflecting increased work-related and activity-related disability. The WPAI has also been validated for use in Greek populations and is available for research applications [33].

Keratoconus severity classification

Disease severity among participants with keratoconus was assessed separately for each eye (OD: right eye; OS: left eye) using the Amsler-Krumeich classification system [30]. This classification categorizes keratoconus into four stages (Stages I-IV) based on refractive error (myopia and astigmatism), mean keratometric values, corneal thickness (pachymetry), and the presence or absence of corneal scarring.

Disease staging was independently verified by a cornea specialist and a professor of ophthalmology to ensure diagnostic accuracy and consistency across evaluations.

Collectively, these instruments provided a multidimensional assessment of the functional, psychosocial, occupational, and economic burden associated with keratoconus and primary open-angle glaucoma, enabling a comprehensive patient-centered evaluation of their real-world impact.

Statistical analysis

All statistical analyses were performed using Statistical Product and Service Solutions (SPSS, version 29.0; IBM SPSS Statistics for Windows, Armonk, NY). Categorical variables are presented as absolute and relative frequencies (%). Normality was assessed prior to parametric testing, and homogeneity of variance was evaluated accordingly. Continuous variables are expressed as mean ± standard deviation (SD) when normally distributed, and comparisons were conducted using independent samples t-tests. For non-normally distributed variables, values are reported as median and interquartile range (IQR), and the Mann-Whitney U test was applied. For each VFQ-25 and WPAI subscale, a two-way ANOVA was conducted with disease group (keratoconus vs. glaucoma) and gender/employment status/type of treatment/comorbidity status as fixed factors, and their interaction term. Statistical significance was set at p < 0.05.

## Results

Demographic characteristics of the sample

The study sample comprised 153 participants, of whom 102 had keratoconus, and 51 had glaucoma (Table 1). Statistically significant differences were observed between the two groups in terms of gender distribution, with males predominating in the keratoconus group (71.6% vs. 43.1%, p < 0.001). Regarding marital status, the majority of keratoconus patients were unmarried (63.6%), whereas glaucoma patients were more frequently married (58.8%, p < 0.001). The two groups also differed significantly in age distribution, as keratoconus patients were considerably younger, with equal proportions across the ≤25, 26-35, and 36+ age categories (33.3% each), while 96.1% of glaucoma patients were aged 36 years or older (p < 0.001). Furthermore, glaucoma patients exhibited a substantially higher prevalence of comorbidities (70.8% vs. 22.4%, p < 0.001).

**Table 1:** Demographic characteristics of the keratoconus and glaucoma subsamples (N = 153) Notes: Values are referred to absolute and relative frequencies (%) and were analyzed using the chi-square test. Statistically significant differences are marked in bold.

Parameters	Keratoconus	Glaucoma	Total	χ^2^	p
Gender					
Male	73 (71.6%)	22 (43.1%)	95 (62.1%)	11.68	<0.001
Female	29 (28.4%)	29 (56.9%)	58 (37.9%)		
Family status					
Married	21 (21.2%)	30 (58.8%)	51 (34.0%)	39.81	<0.001
Unmarried	63 (63.6%)	5 (9.8%)	68 (45.3%)		
Other (divorced, cohabitation)	15 (15.2%)	16 (31.4%)	31 (20.7%)		
Educational level					
Till secondary school	56 (55.4%)	36 (70.6%)	92 (60.5%)	3.25	0.071
Third-level education	45 (44.6%)	15 (29.4%)	60 (39.5%)		
Job					
Economically active population	64 (64.6%)	36 (70.6%)	100 (66.7%)	0.53	0.465
Economically inactive population	35 (35.4%)	15 (29.4%)	50 (33.3%)		
Residence					
Attica	54 (52.9%)	35 (68.6%)	89 (58.2%)	3.44	0.064
Rest of Greece	48 (47.1%)	16 (31.4%)	64 (41.8%)		
Age (years)					
≤25	34 (33.3%)	1 (2.0%)	35 (22.9%)	53.93	<0.001
26-35	34 (33.3%)	1 (2.0%)	35 (22.9%)		
36+	34 (33.3%)	49 (96.1%)	83 (54.2%)		
Type of treatment					
Conservative	58 (58.0%)	35 (68.6%)	93 (61.6%)	1.61	0.204
Surgical	42 (42.0%)	16 (31.4%)	58 (38.4%)		
Comorbidity					
No	76 (77.6%)	14 (29.2%)	90 (61.6%)	31.91	<0.001
Yes	22 (22.4%)	34 (70.8%)	56 (38.4%)		

Subgroup analyses

Keratoconus patients reported significantly better scores across several VFQ-25 domains compared to their glaucoma counterparts (Table 2). Specifically, near activities (70.92 ± 15.86 vs. 61.09 ± 16.76, p < 0.001), distance activities (66.72 ± 17.13 vs. 57.18 ± 19.10, p = 0.002), peripheral vision (71.57 ± 21.98 vs. 52.00 ± 20.10, p < 0.001), and social functioning (p < 0.001) were notably higher in the keratoconus group. Similarly, the VFQ-25 total score was significantly higher among keratoconus patients compared to glaucoma patients (68.98 vs. 61.40, p = 0.004). No statistically significant differences were identified between the two groups in mental health (68.65 ± 17.98 vs. 68.53 ± 19.50, p = 0.969), general vision (37.47 ± 7.17 vs. 37.14 ± 7.42, p = 0.792), or ocular pain (p = 0.370).

**Table 2:** Differences between VFQ-25 and WPAI variables among keratoconus and glaucoma subsamples (N = 153) Notes: Values are referred to mean ± standard deviation (SD) and were analyzed using the t-test or ^‡^ to median, interquartile range, and were analyzed using the Mann-Whitney U test. Statistically significant differences are marked in bold. VFQ-25: Visual Function Questionnaire-25; WPAI: Work Productivity and Activity Impairment Questionnaire

Parameters	Keratoconus	Glaucoma	Total	t/U^‡^	p
VFQ-25					
General Health	35.28 ± 11.81	29.03 ± 10.56	33.20 ± 11.75	3.19	0.002
General Vision	37.47 ± 7.17	37.14 ± 7.42	37.36 ± 7.23	0.26	0.792
Ocular Pain^‡^	75.00 (12.50)	75.00 (12.50)	75.00 (12.50)	2377.0	0.370
Near Activities	70.92 ± 15.86	61.09 ± 16.76	67.64 ± 16.77	3.54	<0.001
Distance Activities	66.72 ± 17.13	57.18 ± 19.10	63.54 ± 18.31	3.12	0.002
Social Functioning^‡^	83.33 (25.00)	66.67 (16.67)	75.00 (25.00)	1585.50	<0.001
Mental Health	68.65 ± 17.98	68.53 ± 19.50	68.61 ± 18.44	0.04	0.969
Role Differences^‡^	75.00 (25.00)	62.50 (18.75)	68.75 (25.00)	2170.50	0.093
Dependency Health^‡^	78.13 (25.00)	81.25 (18.75)	81.25 (25.00)	2456.00	0.571
Driving^‡^	75.00 (25.00)	50.00 (37.50)	66.67 (25.00)	235.50	<0.001
Color Vision^‡^	87.50 (25.00)	75.00 (0.00)	75.00 (25.00)	1430.00	<0.001
Peripheral Vision	71.57 ± 21.98	52.00 ± 20.10	65.13 ± 23.22	5.30	<0.001
Total score^‡^	68.98 (19.56)	61.40 (12.62)	66.05 (18.74)	1866.50	0.004
WPAI					
Absenteeism^‡^	4.76 (6.77)	6.25 (15.69)	5.41 ± 12.12	1112.50	0.108
Presenteeism	44.93 ± 25.46	39.49 ± 21.02	43.00 (24.02)	1.20	0.232
Work productivity loss	49.42 ± 26.87	45.41 ± 20.97	47.99 (24.90)	0.86	0.390
Activity impairment	48.33 ± 26.25	45.10 ± 23.36	47.25 (25.29)	0.75	0.458

No statistically significant differences were observed between the keratoconus and glaucoma groups across any of the WPAI domains, including absenteeism (p = 0.108), presenteeism (p = 0.232), overall work productivity loss (p = 0.390), and activity impairment (p = 0.458), suggesting that the impact on work-related outcomes was comparable between the two conditions.

Two-way ANOVA revealed several significant interaction effects across demographic and clinical subgroups (Table 3). With respect to employment status, economically active participants demonstrated significant group differences in general health (p = 0.002), general vision (p = 0.009), ocular pain (p < 0.001), near activities (p < 0.001), distance activities (p < 0.001), Social Functioning (p = 0.008), mental health (p < 0.001), role difficulties (p = 0.004), dependency (p = 0.002), color vision (p = 0.041), peripheral vision (p < 0.001), and total VFQ-25 score (p < 0.001). Regarding gender, significant differences between keratoconus and glaucoma patients were noted in social functioning (p = 0.028), mental health (p = 0.040), role difficulties (p = 0.029), and dependency (p = 0.012), with keratoconus males generally reporting higher scores. With respect to the type of treatment, surgical patients differed significantly between groups in ocular pain (p = 0.041) and driving (p = 0.013). Finally, concerning comorbidity status, a significant difference was observed in role difficulties between patients with and without comorbidities (p = 0.047).

**Table 3:** Two-way ANOVA of VFQ-25 and WPAI variables across keratoconus and glaucoma subsamples (N = 153) Notes: Values are referred to mean ± standard deviation (SD) and were analyzed using the two-way analysis of variance (ANOVA). F and p-values represent the disease group × (factor) interaction term. Statistically significant differences are marked in bold. VFQ-25: Visual Function Questionnaire-25; WPAI: Work Productivity and Activity Impairment Questionnaire

Parameters		Keratoconus	Glaucoma	F	p
VFQ-25					
General Health	Gender			1.70	0.195
	Male	36.25 ± 11.79	27.95 ± 10.90		
	Female	32.83 ± 11.71	29.84 ± 10.41		
	Job			10.17	0.002
	Economically active	34.47 ± 10.41	32.19 ± 9.72		
	Economically inactive	36.69 ± 13.58	21.43 ± 8.62		
	Type of treatment			0.30	0.584
	Conservative	37.26 ± 11.06	31.37 ± 9.75		
	Surgical	32.00 ± 12.17	23.91 ± 10.74		
	Comorbidity			0.17	0.678
	No	39.25 ± 10.15	37.50 ± 8.13		
	Yes	25.36 ± 6.53	25.18 ± 9.61		
General Vision	Gender			1.74	0.189
	Male	37.92 ± 7.51	36.09 ± 8.06		
	Female	36.33 ± 6.20	37.93 ± 6.94		
	Job			7.11	0.009
	Economically active	37.20 ± 6.57	38.89 ± 6.74		
	Economically inactive	38.34 ± 8.34	32.93 ± 7.50		
	Type of treatment			0.46	0.501
	Conservative	37.74 ± 6.99	37.99 ± 6.27		
	Surgical	36.82 ± 7.51	35.28 ± 9.44		
	Comorbidity			2.00	0.160
	No	38.28 ± 5.90	40.68 ± 6.78		
	Yes	36.59 ± 9.61	35.07 ± 7.17		
Ocular Pain	Gender			1.76	0.186
	Male	69.52 ± 15.45	68.18 ± 21.38		
	Female	63.79 ± 17.15	70.26 ± 13.53		
	Job			21.34	<0.001
	Economically active	64.41 ± 14.24	75.35 ± 11.37		
	Economically inactive	71.79 ± 16.70	55.00 ± 20.49		
	Type of treatment			4.25	0.041
	Conservative	70.47 ± 15.83	75.00 ± 11.34		
	Surgical	64.29 ± 16.23	57.03 ± 21.39		
	Comorbidity			1.37	0.245
	No	70.56 ± 14.82	79.46 ± 11.61		
	Yes	63.07 ± 16.13	64.71 ± 18.07		
Near Activities	Gender			3.78	0.054
	Male	72.26 ± 16.13	57.42 ± 21.40		
	Female	67.53 ± 14.91	63.88 ± 11.80		
	Job			15.79	<0.001
	Economically active	68.88 ± 15.82	66.16 ± 13.30		
	Economically inactive	74.52 ± 15.52	48.94 ± 18.35		
	Type of treatment			<0.01	0.958
	Conservative	74.50 ± 14.50	63.64 ± 15.01		
	Surgical	66.07 ± 16.88	55.52 ± 19.41		
	Comorbidity			<0.01	0.966
	No	74.18 ± 13.49	67.56 ± 11.46		
	Yes	64.20 ± 17.52	57.33 ± 18.02		
Distance Activities	Gender			2.41	0.123
	Male	68.61 ± 17.18	55.36 ± 22.79		
	Female	61.95 ± 16.34	58.56 ± 16.03		
	Job			13.79	<0.001
	Economically active	66.08 ± 16.48	63.21 ± 17.10		
	Economically inactive	69.07 ± 18.12	42.72 ± 15.94		
	Type of treatment			0.59	0.443
	Conservative	69.05 ± 17.34	60.54 ± 18.26		
	Surgical	63.29 ± 16.87	49.84 ± 19.39		
	Comorbidity			0.06	0.801
	No	70.89 ± 15.43	66.31 ± 10.93		
	Yes	57.80 ± 14.15	51.61 ± 19.87		
Social Functioning	Gender			4.91	0.028
	Male	81.37 ± 16.58	64.58 ± 20.32		
	Female	72.70 ± 23.87	70.26 ± 12.18		
	Job			7.15	0.008
	Economically active	77.73 ± 19.48	72.22 ± 13.51		
	Economically inactive	80.71 ± 19.47	57.22 ± 17.85		
	Type of treatment			2.66	0.105
	Conservative	79.68 ± 19.80	71.79 ± 14.22		
	Surgical	77.78 ± 18.92	59.11 ± 17.49		
	Comorbidity			1.10	0.296
	No	83.66 ± 17.45	73.21 ± 10.43		
	Yes	67.46 ± 15.12	63.97 ± 17.04		
Mental Health	Gender			4.28	0.040
	Male	71.87 ± 16.77	67.33 ± 22.58		
	Female	60.56 ± 18.66	69.44 ± 17.17		
	Job			12.63	<0.001
	Economically active	67.91 ± 18.29	74.41 ± 16.20		
	Economically inactive	71.21 ± 16.63	54.42 ± 19.99		
	Type of treatment			1.54	0.217
	Conservative	70.17 ± 17.45	72.29 ± 18.61		
	Surgical	66.49 ± 19.07	60.31 ± 19.45		
	Comorbidity			0.82	0.367
	No	72.60 ± 16.00	79.91 ± 13.77		
	Yes	61.14 ± 16.42	62.39 ± 19.54		
Role Differences	Gender			4.90	0.029
	Male	70.32 ± 18.11	58.24 ± 25.47		
	Female	64.01 ± 17.49	66.52 ± 13.18		
	Job			8.62	0.004
	Economically active	69.37 ± 16.72	68.52 ± 15.56		
	Economically inactive	69.29 ± 17.37	49.58 ± 22.47		
	Type of treatment			0.29	0.589
	Conservative	72.09 ± 18.33	66.79 ± 16.73		
	Surgical	63.44 ± 17.00	54.56 ± 23.36		
	Comorbidity			4.01	0.047
	No	71.16 ± 16.79	77.08 ± 11.26		
	Yes	63.92 ± 19.66	55.88 ± 19.40		
Dependency Health	Gender			6.53	0.012
	Male	80.31 ± 18.09	70.17 ± 27.61		
	Female	69.18 ± 22.34	77.44 ± 15.49		
	Job			9.61	0.002
	Economically active	76.46 ± 19.05	79.86 ± 16.10		
	Economically inactive	80.18 ± 19.91	60.97 ± 27.44		
	Type of treatment			0.76	0.386
	Conservative	79.31 ± 19.91	77.92 ± 19.53		
	Surgical	74.26 ± 20.30	66.41 ± 24.46		
	Comorbidity			0.45	0.506
	No	81.25 ± 17.14	85.71 ± 12.12		
	Yes	69.03 ± 21.17	68.44 ± 23.18		
Driving	Gender			2.28	0.136
	Male	69.33 ± 20.27	43.06 ± 19.41		
	Female	61.11 ± 15.02	52.08 ± 19.80		
	Job			1.84	0.179
	Economically active	67.23 ± 18.36	48.61± 19.65		
	Economically inactive	70.00 ± 26.99	29.17 ± 5.89		
	Type of treatment			6.42	0.013
	Conservative	75.25 ± 12.89	44.79 ± 20.61		
	Surgical	55.95 ± 23.30	54.17 ± 14.43		
	Comorbidity			0.69	0.409
	No	71.63 ± 14.50	44.44 ± 20.83		
	Yes	59.72 ± 29.05	41.67 ± 17.82		
Color Vision	Gender			2.55	0.112
	Male	84.37 ± 19.43	63.64 ± 25.27		
	Female	80.36 ± 19.67	70.69 ± 11.71		
	Job			4.26	0.041
	Economically active	82.03 ± 20.64	70.83 ± 14.02		
	Economically inactive	86.03 ± 17.61	60.00 ± 26.39		
	Type of treatment			1.61	0.206
	Conservative	84.82 ± 18.88	71.43 ± 13.75		
	Surgical	81.55 ± 19.95	59.37 ± 25.62		
	Comorbidity			1.62	0.206
	No	88.16 ± 16.06	73.21 ± 6.68		
	Yes	71.25 ± 20.32	65.44 ± 22.20		
Peripheral Vision	Gender			0.08	0.785
	Male	72.26 ± 22.65	54.55 ± 21.32		
	Female	69.83 ± 20.46	50.00 ± 19.25		
	Job			12.40	<0.001
	Economically active	71.09 ± 21.92	59.29 ± 17.24		
	Economically inactive	73.57 ± 21.81	35.00 ± 15.81		
	Type of treatment			2.18	0.142
	Conservative	74.57 ± 21.71	58.09 ± 19.19		
	Surgical	66.67 ± 21.86	39.06 ± 15.73		
	Comorbidity			1.06	0.305
	No	77.63 ± 18.96	61.54 ± 12.97		
	Yes	55.68 ± 21.73	47.79 ± 21.64		
Total score	Gender			5.33	0.022
	Male	69.13 ± 14.19	57.40 ± 19.36		
	Female	62.33 ± 14.03	62.61 ± 11.32		
	Job			15.47	<0.001
	Economically active	66.47 ± 13.68	65.33 ± 11.98		
	Economically inactive	69.67 ± 14.28	48.44 ± 16.37		
	Type of treatment			0.71	0.401
	Conservative	69.60 ± 14.23	63.58 ± 13.24		
	Surgical	63.77 ± 14.44	53.33 ± 17.67		
	Comorbidity			0.07	0.790
	No	70.98 ± 11.90	68.91 ± 8.58		
	Yes	59.14 ± 14.12	55.67 ± 15.97		
WPAI					
Absenteeism	Gender			0.05	0.822
	Male	9.83 ± 19.82	9.12 ± 7.69		
	Female	13.78 ± 22.12	11.43 ± 10.37		
	Job			1.19	0.278
	Economically active	11.38 ± 21.51	9.72 ± 8.82		
	Economically inactive	7.69 ± 3.99	19.70 ± 12.40		
	Type of treatment			1.52	0.221
	Conservative	8.92 ± 15.86	11.34 ± 9.85		
	Surgical	14.75 ± 26.60	7.16 ± 6.31		
	Comorbidity			1.15	0.286
	No	8.71 ± 15.01	10.36 ± 7.30		
	Yes	18.24 ± 33.52	11.43 ± 10.75		
Presenteeism	Gender			0.21	0.648
	Male	43.20 ± 26.84	38.75 ± 18.57		
	Female	49.05 ± 21.89	40.00 ± 22.96		
	Job			1.44	0.233
	Economically active	46.35 ± 26.05	38.89 ± 21.48		
	Economically inactive	33.75 ± 17.68	46.67 ± 15.28		
	Type of treatment			0.009	0.768
	Conservative	40.00 ± 24.78	37.42 ± 18.79		
	Surgical	53.33 ± 25.12	47.50 ± 28.16		
	Comorbidity			0.07	0.800
	No	42.08 ± 24.76	35.71 ± 18.28		
	Yes	48.67 ± 26.42	45.00 ± 21.33		
Work productivity loss	Gender			0.11	0.743
	Male	47.54 ± 28.16	43.74 ± 19.36		
	Female	53.80 ± 23.65	46.57 ± 22.38		
	Job			1.72	0.192
	Economically active	50.57 ± 27.48	44.48 ± 21.18		
	Economically inactive	39.07 ± 19.01	56.57 ± 17.50		
	Type of treatment			0.30	0.583
	Conservative	44.50 ± 26.52	43.90 ± 19.34		
	Surgical	58.20 ± 26.04	51.26 ± 27.11		
	Comorbidity			0.01	0.938
	No	46.60 ± 25.92	43.03 ± 14.61		
	Yes	53.11 ± 29.34	50.41 ± 22.62		
Activity Impairment	Gender			0.76	0.385
	Male	46.71 ± 28.63	46.36 ± 24.79		
	Female	52.41 ± 18.83	44.14 ± 22.60		
	Job			4.16	0.043
	Economically active	47.19 ± 25.97	39.44 ± 23.17		
	Economically inactive	47.71 ± 25.79	58.67 ± 18.07		
	Type of treatment			0.06	0.806
	Conservative	42.76 ± 26.87	41.43 ± 22.12		
	Surgical	56.67 ± 23.86	53.13 ± 24.69		
	Comorbidity			0.28	0.598
	No	44.74 ± 26.10	35.71 ± 18.28		
	Yes	55.00 ± 24.25	51.18 ± 23.58		

## Discussion

The present cross-sectional study comparatively evaluated VRQoL and work productivity among patients with keratoconus and POAG, two chronic ophthalmic conditions characterized by distinct pathophysiological mechanisms, clinical courses, and demographic profiles. The findings demonstrated that patients with keratoconus reported significantly higher scores across multiple NEI VFQ-25 domains than patients with POAG, whereas no statistically significant differences were observed between the two groups in work productivity outcomes assessed using the WPAI questionnaire. These findings contribute to a better understanding of the differences in patient-reported burden associated with these two ocular disorders.

Regarding demographic characteristics, significant differences were observed between the two study groups, reflecting the well-established epidemiology of the respective diseases. Keratoconus typically affects younger individuals, particularly males, with disease onset occurring during adolescence or early adulthood [3,34,35]. In the present study, 71.6% of patients with keratoconus were male, and participants were relatively evenly distributed across the age groups of ≤25, 26-35, and ≥36 years, findings that are consistent with previous epidemiological reports [5,36]. In contrast, POAG, a condition that predominantly affects older adults, was observed almost exclusively among individuals older than 36 years (96.1%), with a balanced sex distribution [1,37]. The higher prevalence of comorbidities among patients with POAG (70.8% vs. 22.4%, p < 0.001) is likely attributable to the older age profile of this population and is consistent with findings reported in previous studies [8,38].

With respect to VRQoL, the overall VFQ-25 composite score was significantly higher among patients with keratoconus than among those with POAG (68.98 vs. 61.40, p = 0.004). This observation is consistent with the existing literature, which has consistently documented substantial reductions in VRQoL among individuals with glaucoma, particularly in the advanced stages of the disease [39]. Progressive visual field loss, a hallmark of glaucomatous optic neuropathy, has been associated with impairments across several functional domains, including driving ability, face recognition, mobility, and visual performance under low-light conditions [40-42]. Although keratoconus also impairs visual function through irregular astigmatism and progressive refractive distortion, visual performance can often be substantially improved with optical correction or surgical intervention, which may contribute to a comparatively lower functional burden in some patients [43,44].

Analysis of individual VFQ-25 domains further supported these findings. Patients with keratoconus reported significantly better outcomes in near activities (70.92 vs. 61.09, p < 0.001), distance activities (66.72 vs. 57.18, p = 0.002), peripheral vision (71.57 vs. 52.00, p < 0.001), and social functioning (p < 0.001). The markedly lower peripheral vision scores observed among patients with POAG reflect the characteristic pattern of glaucomatous damage, which initially affects peripheral visual field sensitivity before progressing centrally [45]. The extensive impact of glaucoma on functional activities is well documented, with previous studies demonstrating significant impairments in driving performance, independent living, mobility, and participation in social activities [19,46].

Interestingly, no statistically significant differences were observed between the two groups regarding mental health (68.65 vs. 68.53, p = 0.969), general vision (p = 0.792), or ocular pain (p = 0.370). These findings suggest that both diseases exert a comparable burden on these particular dimensions of quality of life. The absence of differences in mental health outcomes may reflect the chronic nature of both conditions, as keratoconus and glaucoma have been associated with increased levels of anxiety, depressive symptoms, psychological distress, and reduced self-esteem [20,47].

Concerning work productivity, no statistically significant differences were identified between patients with keratoconus and those with POAG across any WPAI domain, including absenteeism (p = 0.108), presenteeism (p = 0.232), overall work productivity loss (p = 0.390), and activity impairment (p = 0.458). This finding is noteworthy because it suggests that, despite differences in VRQoL profiles, the two conditions were associated with comparable levels of work-related impairment in the present study population.

Previous studies have documented reductions in work productivity among both patients with keratoconus [48] and individuals with glaucoma [2]. In both conditions, the greatest burden appears to arise from presenteeism rather than absenteeism, indicating reduced effectiveness while at work despite continued attendance. The relatively high presenteeism scores observed in both groups (44.93% and 39.49%, respectively) suggest that chronic visual impairment may substantially compromise workplace performance regardless of the underlying ophthalmic diagnosis. These findings highlight the potential economic consequences associated with chronic ophthalmic diseases for patients, employers, healthcare systems, and society at large [32,48,49].

The two-way ANOVA analyses revealed significant interactions between diagnosis and several demographic and clinical characteristics. Economically active participants demonstrated significant between-group differences across multiple VFQ-25 domains (p < 0.001), potentially because employment requires sustained visual performance and exposes individuals to greater functional demands [50]. A significant interaction between diagnosis and sex was observed for social functioning (p = 0.028), mental health (p = 0.040), and role difficulties (p = 0.029), with male patients with keratoconus reporting more favorable outcomes. These findings may reflect sex-related differences in occupational responsibilities, social expectations, coping strategies, and symptom reporting behaviors [51,52].

Treatment modality was also associated with significant differences in ocular pain (p = 0.041) and driving-related outcomes (p = 0.013). These associations may be attributable to differences in disease severity, indications for surgical intervention, and postoperative visual outcomes [53,54]. Finally, the presence of comorbidities was significantly associated with role difficulties (p = 0.047), suggesting that individuals with multiple chronic health conditions experience cumulative functional limitations that adversely affect daily life [55].

Although patients with POAG demonstrated lower scores in several VRQoL domains compared with patients with keratoconus, these findings should be interpreted within the context of the baseline differences between the two groups. The older age profile, higher comorbidity burden, and differences in treatment characteristics among patients with POAG may have contributed to the observed differences in patient-reported outcomes. Therefore, the findings should be considered as associations between disease groups and patient-reported outcomes rather than definitive evidence of disease-specific effects.

Limitations of the study

Several limitations should be considered when interpreting the findings of this study. First, the cross-sectional design precludes the establishment of causal relationships between disease status and patient-reported outcomes. Second, the unequal sample sizes between the two study groups (102 patients with keratoconus versus 51 patients with POAG) may have influenced the statistical power of certain analyses.

Furthermore, the significant age differences between the two groups represent a potential source of confounding. Although the two-way analysis of variance (ANOVA) allowed the assessment of the effects of demographic and clinical factors, as well as their interactions with disease group, residual confounding cannot be excluded. In addition, differences in treatment status, comorbidity burden, and employment eligibility between the two disease groups may have influenced patient-reported outcomes, particularly work productivity measures. Therefore, the observed differences between patients with keratoconus and those with POAG should be interpreted with caution, as they may partly reflect baseline group heterogeneity rather than the effect of the ophthalmic diagnosis alone. Future prospective studies using cohorts matched for age, sex, and disease severity, together with objective measures of visual function, would provide more robust evidence regarding the comparative burden of these conditions.

Additional limitations include the use of convenience sampling and recruitment from a single tertiary referral center, which may limit the generalizability of the findings to broader populations. Furthermore, the reliance on self-reported questionnaires introduces the possibility of reporting bias and subjective interpretation of disease burden. In addition, the absence of longitudinal follow-up precluded the assessment of changes in VRQoL and work productivity over time.

Another limitation of this study is that glaucoma severity was not classified according to a standardized staging system. Consequently, the independent impact of glaucoma severity on VRQoL and work productivity could not be fully evaluated. Moreover, the multiple subgroup analyses performed in this study increase the risk of type I error due to multiple comparisons. Therefore, these analyses should be considered exploratory and hypothesis-generating rather than definitive, and their findings should be interpreted with caution.

## Conclusions

The present study demonstrates that VRQoL differs significantly between patients with keratoconus and those with POAG, with the latter experiencing greater impairment across several functional domains, including peripheral vision, near and distance activities, driving, and social functioning. In contrast, the impact on work productivity appears comparable between the two groups, highlighting that chronic ophthalmic disease, regardless of its specific etiology, can substantially compromise occupational performance.

These findings emphasize the importance of adopting a holistic approach to patient management that extends beyond conventional clinical treatment and incorporates targeted interventions aimed at functional rehabilitation, psychosocial support, and occupational reintegration. Routine assessment of VRQoL and work productivity using standardized instruments such as the NEI VFQ-25 and WPAI may facilitate more patient-centered clinical care and should be considered an integral component of the management of chronic ophthalmic diseases. Future research should focus on longitudinal investigations examining the evolution of patient-reported outcomes over time and evaluating the effectiveness of therapeutic interventions in improving both functional and socioeconomic outcomes among individuals living with chronic eye disease.
